# Accuracy of vertebral puncture in percutaneous vertebroplasty

**DOI:** 10.1007/s11604-021-01216-3

**Published:** 2021-11-05

**Authors:** Tomoyuki Noguchi, Koji Yamashita, Yoshitaka Shida, Takashi Okafuji, Ryotaro Kamei, Junki Maehara, Tsuyoshi Tajima

**Affiliations:** 1 Department of Radiology, National Hospital Organization Kyushu Medical Center, 1-8-1 Jigyohama, Chuo-ku, Fukuoka City, Fukuoka Province 810-8563 Japan; 2 Department of Clinical Research, National Hospital Organization Kyushu Medical Center, 1-8-1 Jigyohama, Chuo-ku, Fukuoka City, Fukuoka Province 810-8563 Japan; 3 Department of Radiology, Center Hospital, Center for Clinical Sciences, National Center for Global Health and Medicine, 1-21-1 Toyama, Shinjuku-ku, Tokyo 162-8655 Japan; 4 Education and Training Office, Department of Clinical Research, Center for Clinical Sciences, National Center for Global Health and Medicine, 1-21-1 Toyama, Shinjuku-ku, Tokyo 162-8655 Japan

**Keywords:** Vertebroplasty, Punctures, Spine, Needles, Back pain

## Abstract

**Purpose:**

To clarify the accuracy of vertebral puncture of the vertebral tertile area needling (VETERAN) method puncturing the pedicle superimposed on one-third of the width between the lateral vertebral line to the contralateral medial lamina line compared with Cathelin-needle-assisted puncture (CAP) method puncturing using the Cathelin needle as a guide in percutaneous vertebroplasty.

**Materials and methods:**

449 punctures by CAP method and 125 punctures by VETERAN method were enrolled. We compared the puncture accuracy of both methods. We estimated a vertebral estimated tilting ratio (VET-ratio) defined as ratio of the distance between the lateral vertebral line and the contralateral medial laminal line to the distance between the vertebral lateral line and the puncture point measured by computed tomography. We also estimated the procedural items and clinical outcomes.

**Results:**

VETERAN method with 100% of punctures within safe zone (cortical breaches within 2 mm) had significantly higher accuracy than CAP method with 97.8% (*p* < 0.01) for the 2 mm incremental evaluation. No cases with a VET-ratio of 36% or less had cortical breaches. VETERAN method had shorter operative time per puncture (*p* < 0.01) and exposure time per puncture (*p* < 0.05).

**Conclusion:**

VETERAN method reduced the occurrence of the inaccurate puncture, operative times, and exposure times. A VET-ratio with 36% or less is associated with a safe puncture using VETERAN method.

## Introduction

To obliquely puncture the vertebral body through the pedicle has been recommended in percutaneous vertebroplasty (PVP) to avoid the vertebral cortical breaches when puncturing through the transvertebral pedicle parallel to the midline [1]. This oblique puncture, however, is not a simple and easy procedure, and puncture-related complications have been reported; the neurologic complication, the pseudoaneurysm formation due to lumbar injury, the aortic puncture hemorrhage, the spinal epidural, subdural, and subarachnoid hemorrhage [2–7]. Although the frequency of those complications might be low, no respectable studies to investigate the puncture errors have been investigated, neglecting the safety of PVP.

The most common PVP puncture method, the isocenter puncture (ISOP) method [8], is performed as follows. First, the anterior–posterior and lateral projections of the vertebral body are strictly confirmed on fluoroscopy, next the target point of the needle tip placed in the vertebra is set at the isocenter of the fluoroscopy, then the anterior–posterior fluoroscopic view is rotated in the insertion direction, and finally the needle is punctured on-end under fluoroscopy, which is called the bull’s-eye modification method [9], and inserted within the vertebral body through the pedicle. This has a wide range of insertion directions. However, the restless patient during puncturing may need to start over repetitively to get back the target point of the vertebral body to the isocenter.

To cover the shortcomings of bone biopsy needle puncturing using the ISOP method, the Cathelin needle used in the local anesthesia is placed as a guide using ISOP method. The insertion route is planned on the images acquired using the Cone-beam computed tomography (CT) or the interventional CT (IVR-CT), and the needle is punctured in parallel with the Cathelin needle [10–12]. This modification is called the Cathelin-needle-assisted puncture (CAP) method. However, performing CT to plan the insertion route results in the prolonged operative time and exposure time. Another drawback of CAP method was that the Cathelin needle sometimes might not serve as a guide due to displacement, slippage, or overlapping puncture pathways.

We here propose the vertebral tertile area needling method (VETERAN method), which determines the puncture route based on the anatomical structure recognized on the oblique fluoroscopic view without an additional guide device. That is, the anterior–posterior fluoroscopic view is rotated so that the vertebral pedicle is superimposed on one-third of the width between a lateral vertebral line to the contralateral medial lamina line, and then the vertebra is punctured on-end under fluoroscopy using the bull’s-eye modification method [9].

However, VETERAN method as well as CAP method have not been objectively validated for safety or risk. Following the process of establishing quality standards for pedicle screw placement in orthopedics, this study was the first to determine the accuracy of vertebral puncture in percutaneous vertebroplasty by comparing VETERAN and CAP methods.

## Materials and methods

### Study design

This retrospective research was approved by our Institutional Review Board, and the need for written informed consent from each patient was waived based on the retrospective nature of this study.

### Patients

Between May 2015 and January 2019 at our hospital, 563 cases were revealed to have unhealed thoracic or lumbar vertebral fractures on spine CT or MRI. Of those, a PVP was performed for 178 cases, whose preoperative status met the following eligibility criteria: they had (1) one or more unhealed vertebral fractures in the 6th thoracic to 5th lumbar vertebra, (2) severe back pain or a remarkable decrease in activities of daily living (ADLs) due to the vertebral fracture, (3) no active infection, (4) no bleeding diatheses, and (5) they requested a PVP. Of those, 139 cases with 449 punctures made by CAP method from May 2015 to July 2017 (CAP group) and 39 cases with 125 punctures made by VETERAN method from August 2017 to January 2019 (VETERAN group) were enrolled. Demographic information for those two groups was given in Table 1.

**Table 1:** Demographic information of CAP and VETERAN groups

Item	Unit	CAP group	VETERAN group
No. of patients		139	39
No. of puncture	Total (mean per patient)	449 (3.20)	125 (3.28)
Age	Median (range) year	82 (46–97)	80(39–93)
Sex	Male/female	52/87	14/25
Vertebral fracture	No. of patients with 1 to 5 vertebral fractures	89/39/7/3/1	26/9/4/0/0
Neoplastic fracture	No. (%) of patients	4 (3%)	0 (0%)

*No*. Number

### PVP operators and equipment

All procedures were performed in our hospital’s angiographic examination room by two or more radiologists including at least one board-certified interventional radiologist who was familiar with the following PVP procedure using biplane fluoroscopic angiography equipment (Axiom Artis dBA; Siemens Healthcare GmbH, Erlangen, Germany) and interventional CT (IVR-CT) (SOMATOM, Sensation, OPEN; Siemens Healthcare GmbH, Erlangen, Germany).

### PVP procedure

First, the patient was placed in the prone or lateral decubitus position [11] on the examining bed. The area of interest, which was confirmed with fluoroscopy as well as physical examination on the basis of percussion tenderness, was marked and sterilized.

After local infiltration anesthesia was induced, CAP method or VETERAN method was performed. Although the transpedicular approach was usually performed, the transcostovertebral approach for the thoracic vertebra was adopted depending on the operator’s decision. Details of CAP and VETERAN methods were described later.

For patients with large vertebral clefts, the test injection of the carbon dioxide gas were performed to know the space of the cleft prior to bone cement injection.

Bone cement (VertaPlex Bone Cement; Stryker) was slowly injected into the vertebra through the bone biopsy needle(s) using a bone cement injector (PCD System; Stryker, Mahwah, NJ) under continuous fluoroscopy. The injection was terminated when the bone cement adequately diffused in the vertebrae, leaked into extravertebral structures, or migrated into veins. The total maximum dose of the bone cement per patient was limited to 12.5 mL, which is the amount contained in one package of the bone cement product. Prophylactic augmentation to adjacent vertebrae [13] was performed for patients whose current vertebral fracture was caused by events other than falling or trauma, had a widened air- or fluid-filled cleft which seemed to be a mass-like distribution after PVP, or had a concomitant pre-existing compression fracture in another vertebra [14–16]. Pediculoplasty [17] was also performed in addition to PVP by injecting the bone cement along the needling paths to brace the pedicles of fractured vertebrae after confirming by CT that the bone biopsy needle passed through the pedicle on CT. After PVP, the distribution of the bone cement was assessed on IVR-CT, and the patient was then held in a supine or lateral decubitus position for 120 min in the patient’s hospital bedroom.

### CAP method

In CAP method, the Cathelin needle used in the local anesthesia was first placed on the vertebral arch as a guide for the bone biopsy needle using ISOP method. Then, a Cone-beam CT or IVR-CT was performed to plan the puncture routes before puncturing. The choice of the two CT modalities depended on operator’s decision considering the patient’s condition, the number and level of vertebral bodies to be punctured, and the treatment position. If the Cathelin needle might not serve as a guide due to displacement, slippage, or overlapping puncture pathways, the Cathelin needle placement followed by CT scans was repeated. One or more 11- or 13-gauge bone biopsy needles (Osteo-Site Bone Biopsy Needle Set; Cook Medical, Indianapolis, IN) were hammered into the vertebral body by a uni- or bilateral transpedicular or transcostovertebral approach in parallel with the Cathelin needle under fluoroscopic monitoring using the bull’s-eye modification method under fluoroscopy. The Cone-beam CT or IVR-CT was performed again to confirm the insertion route of needles after puncturing.

### VETERAN method

In VETERAN method, the anterior–posterior fluoroscopic view was rotated about 10 to 40 degrees along the body axis so that the vertebral pedicle was superimposed on one-third of the width between a lateral vertebral line to the contralateral medial lamina line as determined by visual judgement on the oblique fluoroscopic view. In the case of transcostovertebral approach for the thoracic vertebra, the costovertebral space was superimposed on one-third of the width between a lateral vertebral line and the contralateral medial lamina line. Then, the bone biopsy needle was hammered into the vertebral body by a uni- or bilateral puncturing with the transpedicular or transcostovertebral approaches using the bull’s-eye modification method under fluoroscopy. A Cone-beam CT or IVR-CT was performed once to confirm the insertion route of the needles after puncturing.

### Estimation of the accuracy of the needle insertion course

To retrospectively assess the accuracy of the needle insertion course in the Cone-beam CT or IVR-CT images acquired after puncturing, the canal encroachment of the needles were measured and classified using Gertzbein and Robbins classification scores which were commonly used for the assessment of the pedicle screw placement in spinal surgery based on CT as follows [18].

Grade A: the screw completely within the pedicle.

Grade B: pedicle cortical breach by 2 mm or less.

Grade C: pedicle cortical breach by 4 mm or less.

Grade D: pedicle cortical breach by 6 mm or less.

Grade E: pedicle cortical breach by more than 6 mm.

**Fig. 1:**
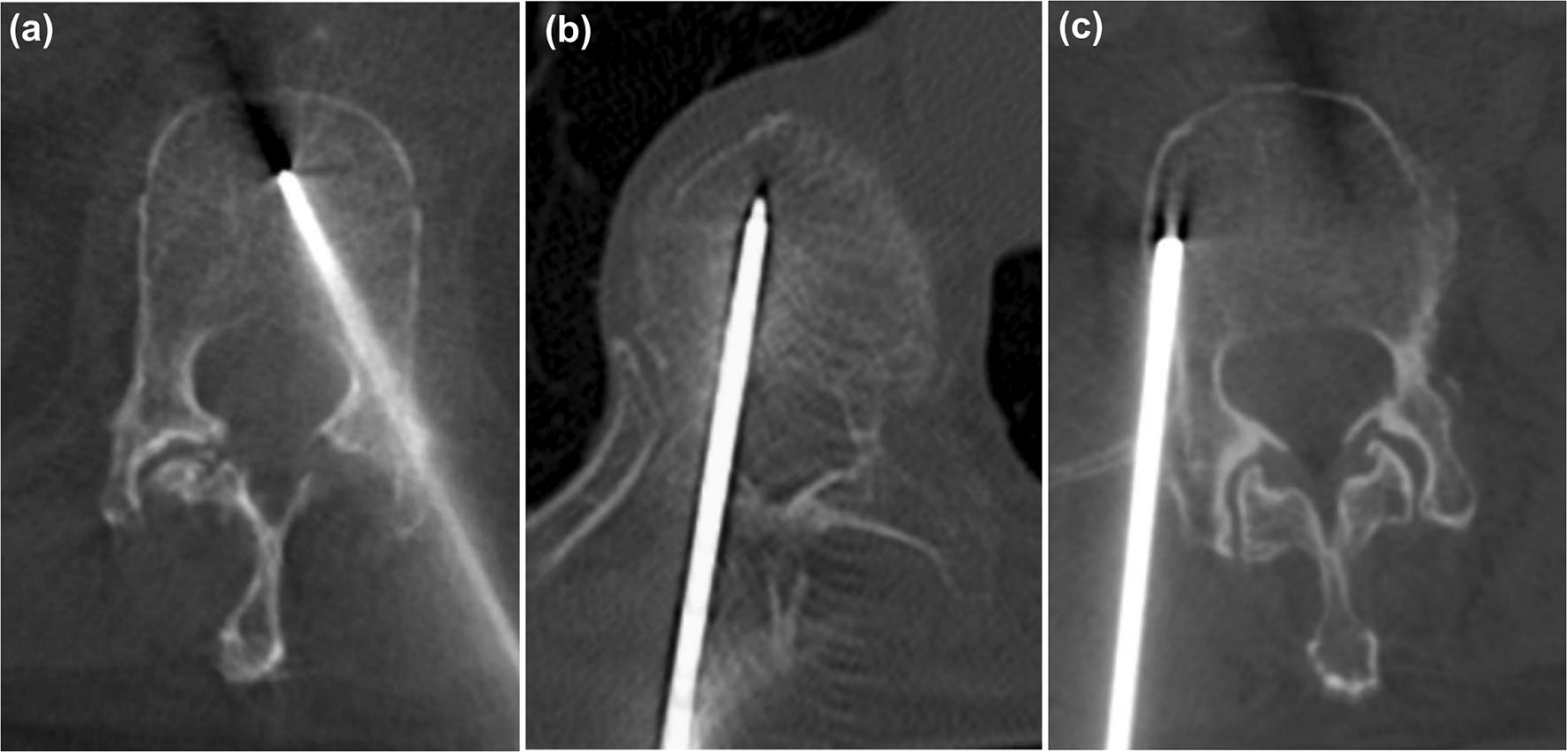
An IVR-CT image (**a**) and Cone-beam CT images (**b** and **c**) of the vertebral body after the bone biopsy needle insertion. Cases **a**, **b**, and **c** are Grade A (no cortical breach) in medial breaches, Grade B (the cortical breach with 2 mm or less) in medial breaches, and Grade C (the cortical breach with 4 mm or less) in lateral breaches, respectively, according to Gertzbein and Robbins classification scores based on CT commonly used to assess the pedicle screw placement in spinal surgery

Grades A and B were considered “clinically acceptable”, and those Grades C-E had a significant deviation from the intended trajectory and were also considered “inaccurate” (Fig. 1) [19]. The difference in the occurrence of the vertebral cortical breaches judged as “inaccurate” or “clinically acceptable” between the VETERAN and CAP groups was determined by the Chi-square test.

**Fig. 2:**
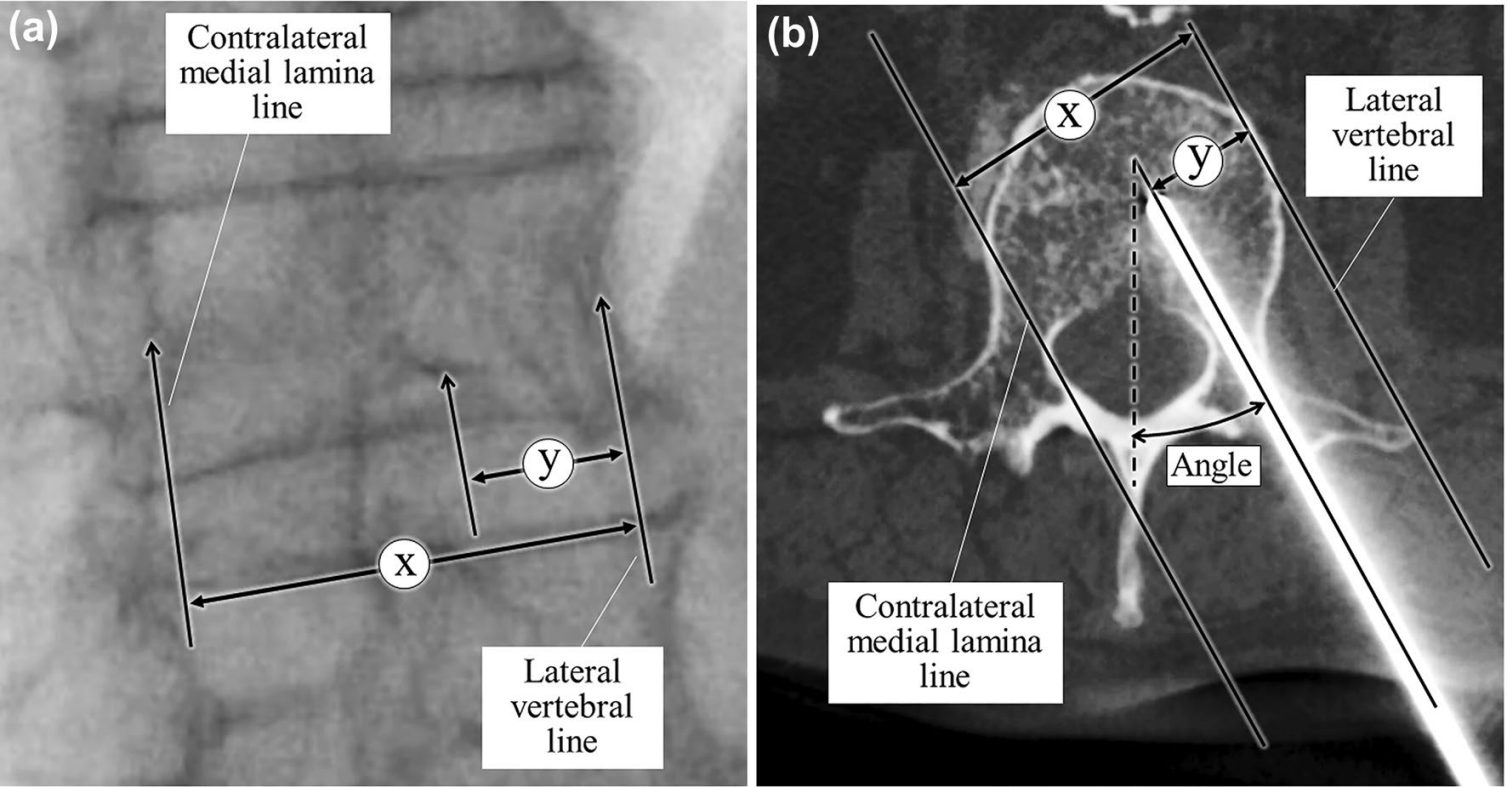
A left anterior oblique fluoroscopic view of the lumbar vertebral body before puncturing (**a**) and an IVR-CT image of the lumbar vertebral body after the insertion of the bone biopsy needle (**b**). The right, center, and left vertical arrows in (**a**) and (**b**) indicate the lateral medial lamina line, the puncture point, and the contralateral vertebral line, respectively. Vertebral estimated tilting ratio (VET-ratio) is equal to y/x (%), where ‘x’ represents the distance between the lateral vertebral line and the contralateral medial lamina line and ‘y’ represents the distance between the vertebral lateral line and the puncture point shown in (**a**) and (**b**). VET-ratio should be 33% in VETERAN method

We measured the puncture angle, which was the inclination towards the midline, as an indicator of safe or warning vertebral puncture. In addition, we also measured the vertebral estimated tilting ratio (VET-ratio) as a surrogate index to the puncture angle. VET-ratio was the distance ratio between a lateral vertebral line to the contralateral medial lamina line and the vertebral lateral line to the puncture point (Fig. 2). The puncture angle and VET-ratio were measured on the Cone-beam CT or IVR-CT images acquired after puncturing.

### Assessment of clinical outcome

Each of the patients performed a self-assessment of his or her back pain on a scale of 0 to 10 (with 0 indicating no pain and 10 indicating the maximum imaginable pain) known as the pain numeric rating scale (NRS) scores [12, 20]. We determined the patients’ mobility ADL scores using the following five-point scale, which is a modification of Yokoyama’s ADL scores [12, 21]: 0 points = complete independence; 1 point = light assistance, being able to walk with walking equipment; 2 points = moderate assistance, needing a wheelchair for locomotion; 3 points = major assistance, mostly staying in bed and being able to sit upright at 60 to 90 degrees; and 4 points = total assistance, mostly staying in a bed-ridden state and being upright under 60 degrees.

The patients’ pain ratings and mobility scores were estimated 1 day before PVP and on the 7th day after PVP. If there were any lost or missing score data 7 days after PVP, we used the scores that were rated closest to the date of the lost data. We also assessed post-PVP complications and adverse events among the patients. All data were identified using our institution’s Hospital Information System.

We estimated the differences between CAP and VETERAN groups with regard to other items including the procedural success rates, the number of the uni- or bilateral puncture, occurrence of bone cement leakage, pulmonary emboli, and cardiac dysfunction by the Chi-square test, the operative time per puncture and radiation exposure time per puncture, Cone-beam CT exposure dose, the number of Cone-beam CT scans, IVR-CT exposure dose using the dose length product (DLP), the number of IVR-CT scans, the bone cement volume per vertebra, pain NRS scores at 1 day before PVP and on the 7th day after PVP, and mobility ADL scores at 1 day before PVP and on the 7th day after PVP by the Mann–Whitney’s *U* test.

### Statistical analysis

The statistical analysis was properly performed using Excel 2013 (Microsoft, Seattle, WA) with an add-in software Statcel-3 [22], regarding the correlation analysis using Pearson’s correlation coefficient test, the univariate analysis using Mann–Whitney’s *U* test or the Chi-square test. The level of significance was set at *P* < 0.05 for all tests.

## Results

### Estimation of the accuracy of the needle insertion course

Table 2 showed the number distribution of the punctures of Gertzbein and Robbins classification scores for CAP and VETERAN groups. The “clinically acceptable “accuracy of needle puncture (Grades A and B) in CAP and VETERAN groups was 97.8% and 100%, respectively. Statistical analysis showed VETERAN group had statistically significant superiority (*P* < 0.01) in “clinically acceptable” accuracy compared with CAP group.

**Table 2:** Number distribution of the punctures of Gertzbein and Robbins classification scores for CAP and VETERAN groups

Vertebral Level	Gertzbein and Robbins classification score										
	CAP group							VETERAN group			
	Grade A	Grade B in medial breach	Grade C in medial breach	Grade D in medial breach	Grade B in lateral breach	Grade C in lateral breach	TCT	Grade A	Grade B in medial breach	Grade B in lateral breach	TCT
T5								1			
T6	2										
T7	5						1	1			
T8	8										
T9	9						1				1
T10	14	4					4	2			
T11	39						7	9			1
T12	54	3	1				7	17	1		2
L1	93	2	2		4	1		34		1	
L2	71	1	1		1	3		28		1	
L3	63			1	2			21			
L4	27	1				1		4			
L5	15	1						1			
Total (%)	89.1%	2.7%	0.9%	0.2%	1.6%	1.1%	4.5%	94.4%	0.8%	1.6%	3.2%

*T* thoracic vertebra, *L* lumbar vertebra, *TCT* transcostovertebral approach

Table 3 showed the averages and ranges of the needle puncture angles for CAP and VETERAN groups. When CAP and VETERAN groups were combined, the ranges of the puncture angles in Grade A, the medial cortical breaches in Grade B-D, and the lateral cortical breaches in Grade B–C were 3–42, 14–40, and 9–33 degrees, respectively.

**Table 3:** Average (range) of the needle puncture angle for CAP and VETERAN groups

Vertebral Level		Gertzbein and Robbins classification score																			
		CAP group													VETERAN group						
		Grade A		Grade B in medial breach		Grade C in medial breach		Grade D in medial breach		Grade B in lateral breach		Grade C in lateral breach		TCT	Grade A		Grade B in medial breach		Grade B in lateral breach		TCT
T5															23						
T6	9 (8–10)												0								
T7	11 (3–18)												34		7						
T8	19 (9–27)																				
T9	15 (3–31)												16							35	
T10	22 (7–34)		27 (18–38)										27 (16–39)		24 (23–25)						
T11	22 (10–40)												22 (6–31)		24 (12–30)					39	
T12	22 (5–39)		23 (14–28)		27								25 (13–30)		20 (12–28)	24				30 (24–35)	
L1	22 (6–39)		23 (14–31)		25 (22–28)				21 (12–33)		15				19 (5–30)			14			
L2	22 (12–35)		28		40				23		15 (12–20)				22 (11–30)			15			
L3	24 (8–38)						31		14 (9–18)						19 (4–28)						
L4	28 (11–42)		39								15				18 (15–25)						
L5	30 (20–38)		38												29						
Total	23 (3–42)		27 (14–39)		29 (22–40)		31		19 (9–33)		15 (12–20)		25 (6–39)		20 (4–30)	24		14 (14–15)		27 (22–33)	

*T* thoracic vertebra, *L* lumbar vertebra, *TCT* transcostovertebral approach

Table 4 showed the averages and ranges of the VET-ratios for CAP and VETERAN groups. When CAP and VETERAN groups were combined, the ranges of VET-ratios in Grade A, the medial cortical breaches in Grade B-D, and the lateral cortical breaches in Grade B-C were 4–50%, 37–63%, and 6–35%, respectively.

**Table 4:** Average (range) of VET-ratio for CAP and VETERAN groups

Vertebral Level	Gertzbein and Robbins classification score										
	CAP group							VETERAN group			
	Grade A	Grade B in medial breach	Grade C in medial breach	Grade D in medial breach	Grade B in lateral breach	Grade C in lateral breach	TCT	Grade A	Grade B in medial breach	Grade B in lateral breach	TCT
T5								30			
T6	22 (22–22)										
T7	26 (4–34)						38	22			
T8	33 (25–44)										
T9	33 (26–44)						43				39
T10	34 (12–49)	59 (58–61)					34 (17–43)	38 (38–39)			
T11	33 (17–48)						28 (16–39)	33 (24–46)			42
T12	31 (16–50)	43 (37–50)	56				28 (20–39)	31 (25–36)	39		34 (32–36)
L1	33 (7–50)	46 (42–51)	51 (50–52)		26 (20–35)	6		32 (19–43)		16	
L2	35(19–50)	40	61		23	18 (12–21)		34 (26–42)		17	
L3	33(16–47)			63	12 (8–17)			31 (19–43)			
L4	30 (16–48)	56				6		26 (20–31)			
L5	22 (6–32)	53						24 (24–24)			
Total	32 (4–50)	51 (37–61)	55 (50–61)	63	22 (8–35)	13 (6–21)	31 (16–46)	32 (19–46)	39	17 (16–17)	33 (24–39)

*T* thoracic vertebra, *L* lumbar vertebra, *TCT* transcostovertebral approach

### Procedural items and clinical outcomes

Table 5 showed the results of the procedural items and clinical outcomes. Both CAP and VETERAN groups had 100% procedural success rates. The VETERAN group had significantly shorter operative time per puncture (with the mean ± SD being 29 ± 8 vs. 24 ± 9, respectively, *p* < 0.01) and shorter exposure time per puncture (with the mean ± SD being 10 ± 3 vs. 8.8 ± 3.4, respectively, *p* < 0.05) than CAP group. In the VETERAN group, the Cone-beam CT exposure dose was significantly lower (with the mean ± SD being 352 ± 173 vs. 155 ± 83 mGy, respectively, *p* < 0.01), and the number of Cone-beam CT scans was smaller (with the mean (range/median) being 2.1 (0–4/2) vs. 0.9 (0–2/1) times, respectively, *p* < 0.01), compared with the CAP group, while there were no differences observed in the IVR-CT exposure dose or the number of IVR-CT scans.

**Table 5:** Results of the procedural items and clinical outcomes

Items	Unit	CAP group	VETERAN group	*p* value
Procedural success rate	%	100%	100%	> 0.999
Operative time	Mean ± SD minutes per puncture	29 ± 8	24 ± 9	< 0.01
Exposure time	Mean ± SD minutes per puncture	10 ± 3	8.8 ± 3.4	< 0.05
Cone-beam CT exposure dose	Mean ± SD mGy	352 ± 173	155 ± 83	< 0.01
No. of Cone-beam CT	Mean(Range/Median)	2.1 (0–4/2)	0.9 (0–2/1)	< 0.01
IVR-CT exposure dose (DLP)	Mean ± SD mGy	656 ± 331	617 ± 306	0.323
No. of IVR-CT	Mean(Range/Median)	1.1 (1–3/1)	1.2 (1–2/1)	0.062
Uni-or bilateral puncture	No. of uni-/bilateral puncture	133/158	49/38	0.082
Bone cement volume	Mean(range/median) mL per vertebra	1.6 (0–7/1.4)	1.8 (0.4–6/1.6)	> 0.999
Bone cement leakage	No. and (%) of vertebra	168(58%)	62(71%)	0.194
Pulmonary emboli	No. and (%) of pts	3 (2%)	0 (0%)	0.697
Cardiac dysfunction	No. and (%) of pts	3 (3%)	0 (0%)	0.818
NRS score before PVP	Mean ± SD	6.4 ± 3	6.6 ± 3	0.818
NRS score at 7th day	Mean ± SD	2.3 ± 2.4	2.8 ± 2.1	0.71
ADL score before PVP	Mean ± SD	2.7 ± 1.3	2.8 ± 1.4	0.962
ADL score at 7th day	Mean ± SD	1.5 ± 1	1.4 ± 1	0.765

*SD* standard deviation, *CT* computed tomography, *DLP* dose length product, *IVR*-*CT* interventional computed tomography, *No*. number, *Pts* patients, *NRS* numeric rating scaling, *ADL* activities of daily living

No difference was observed between the two groups in the number of uni- or bilateral puncture, bone cement volume, the development of bone cement leakage, pulmonary embolism, cardiac dysfunction, pain NRS, or mobility ADL.

## Discussion

In addition to ISOP method and CAP method, the bull’s-eye modification method, the angle fixation method using fluoroscopy, or CT-guided puncture have been proposed to improve the accuracy of the vertebral puncture in PVP [2, 8, 9, 23, 24]. All of the several PVP puncture methods have advantages and challenges. The bull’s-eye modification method is to insert the needle so that it is viewed on-end via fluoroscopy so as to ensure straightness in the insertion direction. This is the basic needling method performed using fluoroscopy and it is easy to implement. However, this does not help determine the puncture course. The fixed angle insertion method involves inserting the needle at a fixed angle (15–25 degrees) and puncturing from the outside to the inside of the vertebral pedicle in the anterior–posterior fluoroscopic view. This method is also simple, but it limits the direction at which the needle can be inserted. CT-guided puncture can assist the safe and accurate puncturing into the vertebra, but the CT device is occupied for PVP for 2 to 3 h. Anyway, there is no reliable CT-based safety assessment for PVP vertebral puncture with any method.

Numerous studies have been reported in orthopedics regarding the safety assessment of the pedicle screw placement. Aoude AA et al. conducted a systematic review of pedicle screw placement accuracy in spine surgery. They reported that the freehand or fluoroscopic techniques and navigation had the averaged accuracies of 91.4% and 97.3%, respectively, for Grades A and B pedicle screws placement estimated using Gertzbein and Robbins classification scores [25]. In our study, Grades A and B needle punctures in CAP and VETERAN groups were 97.8% and 100%, respectively, which both methods might have “clinically acceptable” accuracies of the vertebral punctures. In particular, VETERAN method significantly had the effect of reducing the “inaccurate” vertebral puncture as well as shortening the operative and exposure times per puncture compared with CAP method. This is because VETERAN method did not use Cone-beam CT to plan the puncture route before puncturing. Therefore, we recommend VETERAN method when puncturing the vertebrae using the fluoroscopy technique.

The ranges of the puncture angles with medial and lateral breaches both were overlapped with those of Grade A puncture angle in CAP and VETERAN groups combined in our clinical results shown in Table 3. Therefore, the puncture angle might not be always a good indicator of safe vertebral puncture.

According to our results shown in Table 4, there were no cases of medial cortical breaches when the VET-ratio was 36% or less, but there were some cases of medial cortical breaches when the VET-ratio was 37–50%. A VET-ratio of over 50% could be dangerous in all cases in response to the medial cortical breaches. Therefore, 36%, 37% and 50% have been defined as the bounds for safe, cautionary, or dangerous VET ratios, respectively. These bounds of VET-ratio might help prevent the spinal canal intrusion. On the other hand, the safe lower limit of VET-ratio was difficult to determine in our results. It is necessary to find another index related to lateral cortical breaches in the further investigation. In addition, VETERAN has a potential risk of human error because it is judged only by visual measurement. Puncture training is indispensable to avoid this risk.

Our leakage rates (CAP: 58%, VETERAN: 71%) did not exceed 72–91% reported in previous articles [26, 27]. However, further measures such as improved cement properties or the injection volume rates should be considered to reduce the leakage.

Various leading-edge puncture support devices have been developed. Xu HT et al. proposed a navigation system that supported correct puncturing in PVP working with the C-arm tracker, patient tracker, and puncture needle tracker adjusting the spinal image information [28]. Wang B et al. proposed a method for guiding punctures in Balloon kyphoplasty (BKP) using a system consisting of a reference tracker, a robotic arm, and a monitor [29]. As a unique attempt, the 3D-printer was used to create a puncture guide adapter for PVP, which had two sockets for inserting needles [30]. Those puncture methods are promising methods and further development is desired in the future.

This study has some limitations. VET-ratio was not measured on the actual fluoroscopic images. The effectiveness of visual VET-ratio on a real-time fluoroscopy has not been investigated. This study was performed in a retrospective fashion with a small number of punctures. The difference in skill levels among the operators was not considered. A prospective study involving relatively uniform experts and a large number of patients is desired.

In conclusion, this was the first study to determine the accuracy of vertebral puncture to help establish quality standards of the vertebral puncture in percutaneous vertebroplasty. The accuracies of vertebral punctures by CAP and VETERAN methods accounted for 97.8% and 100% of within safe zone (cortical breaches within 2 mm), respectively, indicating that both had “clinically acceptable” accuracies according to Gertzbein and Robbins classification scores. VETERAN method had the significant benefits of shortening the operative and exposure times and reducing the occurrence of the inaccurate vertebral puncture compared with CAP method. In the case of vertebral puncture by VETERAN method, safe puncture is possible with a VET-ratio of 36% or less, caution is required with a VET ratio of 37% or more, and VET ratio must not exceed 50% during puncture.

## Abbreviations

ADL: Activities of daily living
CAP: Cathelin-needle-assisted puncture
CT: Computed tomography
ISOP: Isocenter puncture
IVR-CT: Interventional computed tomography
NRS: Numeric rating scaling
PVP: Percutaneous vertebroplasty
SD: Standard deviation
VETERAN: Vertebral tertile area needling
VET-ratio: Vertebral estimated tilting ratio
Vs.: Versus
